# Response to Letter to the Editor on “Causal associations between prostate diseases, renal diseases, renal function, and erectile dysfunction risk: a 2-sample Mendelian randomization study”

**DOI:** 10.1093/sexmed/qfae059

**Published:** 2024-09-15

**Authors:** Diliyaer Dilixiati, Kaisaierjiang Kadier, Jian-De Lu, Shiping Xie, Baihetiya Azhati, Reyihan Xilifu, Mulati Rexiati

**Affiliations:** Department of Urology, First Affiliated Hospital of Xinjiang Medical University, Urumqi 830054, China; Department of Cardiology, First Affiliated Hospital of Xinjiang Medical University, Urumqi 830054, China; Department of General Surgery, Children's Hospital of Xinjiang Uygur Autonomous Region, Urumqi 830054, China; Graduate School of Xinjiang Medical University, Xinjiang Uygur Autonomous Region, Urumqi 830054, China; Department of Urology, First Affiliated Hospital of Xinjiang Medical University, Urumqi 830054, China; Department of Urology, First Affiliated Hospital of Xinjiang Medical University, Urumqi 830054, China; Department of Nephrology, First Affiliated Hospital of Xinjiang Medical University, Urumqi 830054, China; Department of Urology, First Affiliated Hospital of Xinjiang Medical University, Urumqi 830054, China

First and foremost, we extend our sincere gratitude to Zhang et al for their dedication to advancing scientific knowledge. True scientific progress is achieved through iterative research, critical discourse, and persistent pursuit of accurate solutions. We are deeply inspired by the commitment demonstrated by Zhang et al and aspire to emulate their dedication in our future endeavors. It is imperative to acknowledge, however, that while we hold great respect for the perspectives presented by Zhang et al, endorsing them may pose challenges. Whether considering contemporary methodological standards or the findings of previously published Mendelian randomization (MR) studies, convincing a broad audience presents inherent complexities. Their letter lacks sufficient evidence to support their views and urges us, along with many other researchers who have published ED-related MR studies, to reconsider and update our methodologies and article content.

To address Zhang et al’s inquiry, we performed a thorough search on PubMed using the term “Mendelian randomization and erectile dysfunction,” yielding a total of 47 relevant articles. Of the 47 articles examined, 1 was our original publication, 2 were Chinese articles, and 2 were letters. Additionally, the original erectile dysfunction (ED) genome-wide association study by Bovijn et al^1^ constituted one of the articles. Among the remaining 41 articles, 85.6% utilized meta-analysis data from Bovijn et al as their primary data source, including 2 articles published in *Sexual Medicine*. None of the studies employed the MRlap method for analysis. Each of these studies was meticulously reviewed, and we are prepared to offer a comprehensive overview of the literature sources pertinent to the investigation of ED. For clarity and reference, we have compiled the findings into Table 1, which we are eager to share. Additionally, although reverse MR is deemed essential, it is unfeasible with the data from Bovijn et al. This limitation arises because only 1 single nucleotide polymorphism remains as an instrumental variable postremoval of data affected by linkage disequilibrium. Consequently, reverse MR cannot be conducted. Table 1 provides detailed doi information for MR studies on ED utilizing this dataset. Most of these studies have not executed reverse MR. It is possible to use the Web tool MRbase to assess the suitability of this data for reverse MR analysis. We anticipate that the findings will corroborate our results. This restriction was explicitly acknowledged in our original article’s Limitations, signifying the current inability to pursue reverse MR.

**Table 1:** Current published articles on Mendelian randomization related to ED.

Title	Author	Year	Exposure	Outcome	doi	Data Source for ED
Insights into modifiable risk factors of erectile dysfunction, a wide-angled Mendelian randomization study	Yang Xiong	2024	42 predominant risk factors	ED	10.1016/j.jare.2023.05.008	Bovijn et al^1^
The association between the gut microbiota and erectile dysfunction	Tianle Zhu	2024	Gut microbiota, ED	ED, gut microbiota	10.5534/wjmh.230181	Bovijn et al^1^
Causal association between JAK2 and erectile dysfunction: a Mendelian randomization study	Yu-Jia Xi	2023	JAK2	ED	10.1186/s12610-023-00192-0	Other
A drug target for erectile dysfunction to help improve fertility, sexual activity, and wellbeing: mendelian randomisation study	Benjamin Woolf	2023	PDE5	Pulmonary hypertension, ED	10.1136/bmj-2023-076197	Other
Genetically predicted insomnia causally increases the risk of erectile dysfunction	Yang Xiong	2023	Sleep traits	ED	10.4103/aja202261	Bovijn et al^1^
Genetic susceptibility to COVID-19 may increase the risk of erectile dysfunction: a two-sample Mendelian randomization study	Kun Zhang	2022	Susceptibility to COVID-19	ED	10.1111/and.14527	Bovijn et al^1^
Type 2 diabetes mellitus increases risk of erectile dysfunction independent of obesity and dyslipidemia: a Mendelian randomization study	Chi Yuan	2022	Metabolic syndrome	ED	10.1111/andr.13132	Bovijn et al^1^
Effects of obesity-related anthropometric indices and body composition on erectile dysfunction mediated by coronary artery disease: a Mendelian randomization study	Binghao Bao	2024	Obesity-related anthropometric indicators/body composition	ED	10.1111/andr.13443	Bovijn et al^1^
Causal association of cardiovascular disease with erectile dysfunction: a two-sample bidirectional Mendelian randomization analysis	Miaoyong Ye	2023	Cardiovascular diseases, ED	Cardiovascular diseases, ED	10.1111/andr.13421	Bovijn et al^1^
Causal relationship between worry, tension, insomnia, sensitivity to environmental stress and adversity, and erectile dysfunction: a study using Mendelian randomization	Hao Zhang	2023	ED, psychological states	ED, psychological states	10.1111/andr.13574	Bovijn et al^1^
A Mendelian randomization study on causal effects of leisure sedentary behavior on the risk of erectile dysfunction	Zhao Huangfu	2024	Leisure sedentary behavior	ED	10.1111/andr.13611	Bovijn et al^1^
Causal effects of gut microbiota on the risk of erectile dysfunction: a Mendelian randomization study	Ran Xu	2024	Gut microbiota	ED	10.1038/s41443-024-00824-7	Bovijn et al^1^
Thyroid function, sex hormones and sexual function: a Mendelian randomization study	Alisa D. Kjaergaard	2021	Thyroid function	ED	10.1007/s10654-021-00721-z	Other
Causal association between cardiovascular diseases and erectile dysfunction, a Mendelian randomization study	Qingying Li	2023	Cardiovascular diseases	ED	10.3389/fcvm.2023.1094330	Bovijn et al^1^
Causal effects of hypertension on risk of erectile dysfunction: a two-sample Mendelian randomization study	Zheng Wang	2023	Hypertension	ED	10.3389/fcvm.2023.1121340	Bovijn et al^1^
Genetically predicted hypertension, antihypertensive drugs, and risk of erectile dysfunction: a Mendelian randomization study	Cong Zhao	2023	Hypertension, antihypertensive drugs	ED	10.3389/fcvm.2023.1157467	Bovijn et al^1^
Genetic evidence supporting a causal role of snoring in erectile dysfunction	Yang Xiong	2022	Snoring	ED	10.3389/fendo.2022.896369	Bovijn et al^1^
Specific gut microbiota may increase the risk of erectile dysfunction: a two-sample Mendelian randomization study	Quanxin Su	2023	Gut microbiota	ED	10.3389/fendo.2023.1216746	Bovijn et al^1^
Type 2 diabetes mellitus and the risk of male infertility: a Mendelian randomization study	Xiao-Bin Zhu	2023	Type 2 diabetes mellitus	ED	10.3389/fendo.2023.1279058	Bovijn et al^1^
Potential causal association between aspirin use and erectile dysfunction in European population: a Mendelian randomization study	Rongkang Li	2024	Aspirin usage	ED	10.3389/fendo.2023.1329847	Bovijn et al^1^
Genetic association of lipid-lowering drug target genes with erectile dysfunction and male reproductive health	Quanxin Su	2024	Potential impact of Lipid-lowering drug targets	ED	10.3389/fendo.2024.1362499	Bovijn et al^1^
Genetic evidence suggests that depression increases the risk of erectile dysfunction: a Mendelian randomization study	Kai Ma	2022	Depression	ED	10.3389/fgene.2022.1026227	Bovijn et al^1^
Genetic liability to inflammatory bowel disease is causally associated with increased risk of erectile dysfunction: evidence from a bidirectional Mendelian randomization study	Renbing Pan	2024	Inflammatory bowel disease, ED	Inflammatory bowel disease, ED	10.3389/fgene.2024.1334972	Other
Inflammatory cytokine profiles in erectile dysfunction: a bidirectional Mendelian randomization	Dongze Liu	2024	Inflammatory cytokine profiles, ED	Inflammatory cytokine profiles, ED	10.3389/fimmu.2024.1342658	Other
Mendelian randomization study reveals the effect of idiopathic pulmonary fibrosis on the risk of erectile dysfunction	Kun Zhang	2023	Idiopathic pulmonary fibrosis	ED	10.3389/fmed.2023.1162153	Bovijn et al^1^
Causal effects of gut microbiota on erectile dysfunction: a two-sample Mendelian randomization study	Yuyang Zhang	2023	Gut microbiota	ED	10.3389/fmicb.2023.1257114	Bovijn et al^1^
Association between atorvastatin and erectile dysfunction: a comprehensive analysis incorporating real-world pharmacovigilance and Mendelian randomization	Kaiqin Chen	2024	Atorvastatin	ED	10.3389/fphar.2024.1382924	Bovijn et al^1^
Causal associations between educational attainment and 14 urological and reproductive health outcomes: a Mendelian randomization study	Menghua Wang	2021	Educational attainment	ED	10.3389/fpubh.2021.742952	Bovijn et al^1^
Genetically proxied intestinal microbiota and risk of erectile dysfunction	Fuxun Zhang	2024	Gut microbiota, ED	Gut microbiota, ED	10.1111/andr.13534	Bovijn et al^1^
Assessing the causal relationship between COVID-19 and post-COVID-19 syndrome: a Mendelian randomisation study	Yiming Tao	2023	COVID-19	ED	10.7189/jogh.13.06054	Bovijn et al^1^
Association between genetically proxied HMGCR inhibition and male reproductive health: a Mendelian randomization study	Zhaoqi Yan	2023	Statin use	ED	10.1097/MD.0000000000034690	Bovijn et al^1^
Relationship between inflammatory bowel disease and erectile dysfunction: a 2-sample Mendelian randomization study	Dawei Gao	2024	Inflammatory bowel disease	ED	10.1093/sexmed/qfad067	Bovijn et al^1^
Genetic prediction of modifiable lifestyle factors for erectile dysfunction	Yu-Jia Xi	2024	Lifestyle factors	ED	10.1093/sexmed/qfae010	Bovijn et al^1^
Genetically predicted cardiovascular diseases could increase the risk of erectile dysfunction: a bidirectional Mendelian randomization	Yujia Xi	2023	Cardiovascular diseases, ED	Cardiovascular diseases, ED	10.1007/s00345-023-04630-6	Bovijn et al^1^
Evaluation of bi-directional causal association between periodontal disease and erectile dysfunction: a two-sample Mendelian randomization study	Feiyan Yu	2023	Periodontal disease, ED	Periodontal disease, ED	10.1007/s00784-023-05201-0	Bovijn et al^1^
The association between lipid parameters and erectile dysfunction: a two-sample Mendelian randomization and case–control study	Minghui Ke	2023	Lipid parameters	ED	10.1007/s12020-023-03653-8	Bovijn et al^1^
Effects of heart failure and coronary artery disease on erectile dysfunction: a two-sample mendelian randomization study	Kaiyang Shao	2023	Heart failure, coronary artery disease	ED	10.1186/s12894-023-01335-1	Bovijn et al^1^
Effects of major depression and bipolar disorder on erectile dysfunction: a two-sample mendelian randomization study	Wei-Kang Chen	2023	Depression, bipolar disorder	ED	10.1186/s12920-023-01498-8	Bovijn et al^1^
Association of high LDL concentrations with erectile dysfunction from a Mendelian randomization study	Quan Zhu	2023	Lipid-lowering drugs	ED	10.1038/s41598-023-49771-1	Bovijn et al^1^
A Mendelian randomization study on causal effects of inflammatory bowel disease on the risk of erectile dysfunction	Di Chen	2024	Inflammatory bowel disease	ED	10.1038/s41598-024-52712-1	Other
The association between serum 25-hydroxyvitamin D levels and erectile dysfunction: a two-sample Mendelian randomization analysis	Dawei Gao	2024	Serum 25-hydroxyvitamin D levels	ED	10.1038/s41443-024-00862-1	Bovijn et al^1^

A total of 85.4% of the studies employed data from the same source as our ED genome-wide association study.

Abbreviation: ED, erectile dysfunction.

Zhang et al referred to MRlap, a novel MR tool aimed at addressing the “sample overlap bias” issue. However, it is important to note that, initially, we consulted recent guidelines^2^ and checklists^3^ for MR. According to current guidelines, MRlap is not required for a standardized MR study, unlike the Cochran’s Q statistic, which assesses heterogeneity, or MR-Egger regression, which is used to account for pleiotropy. Current guidelines do not mandate the use of MRlap. Furthermore, a simple PubMed search for “MRlap” and “Mendelian randomization” yielded only 8 results. The presentation method cited in Zhang et al was published in June 2023,^4^ whereas the first article employing this method was published in November 2023. Our original article was submitted to the journal in November 2023. As frontline clinicians, what warrants the adoption of this approach when guidelines lack clarity, there is a dearth of published articles, and no high-quality, high-impact factor journals have endorsed it? Has this approach undergone adequate validation to establish its validity and scientific rigor?

**Figure 1:**
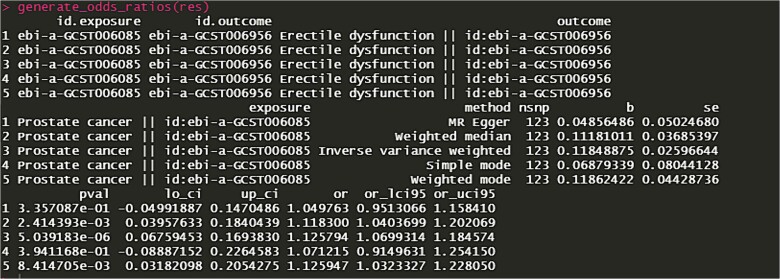
Mendelian randomization results obtained without employing PhenoScanner to mitigate confounding factors (Mendelian randomization analysis indicating the sole positive impact of prostate cancer on erectile dysfunction).

An integral aspect of 2-sample MR is mitigating confounding factors, as outlined in our original article. To ensure that exposure factors do not skew results through confounding, we extensively reviewed guidelines for ED and published Mendelian randomization studies, employing the PhenoScanner tool for confounder exclusion. PhenoScanner is widely utilized and endorsed,^5^^-^^6^ with comprehensive interpretation available in the MR dictionary of the University of Bristol.

Furthermore, we meticulously detailed single nucleotide polymorphism exclusions and rationales in the original Supplementary Material. Even without PhenoScanner, subsequent MR analyses yielded consistent results, underscoring the stability of our findings. For verification, it is possible to explore the MRbase webpage, an official tool providing direct access to results (Figure 1).

## Data Availability

All data in our Mendelian randomization analyses are available from public databases (https://gwas.mrcieu.ac.uk/).
